# Iron status determined changes in health measures induced by nordic walking with time-restricted eating in older adults– a randomised trial

**DOI:** 10.1186/s12877-024-04876-8

**Published:** 2024-03-29

**Authors:** Jakub Antoni Kortas, Joanna Reczkowicz, Ulana Juhas, Ewa Ziemann, Aleksandra Świątczak, Katarzyna Prusik, Szczepan Olszewski, Nakisa Soltani, Ewa Rodziewicz-Flis, Damian Flis, Małgorzata Żychowska, Grażyna Gałęzowska, Jędrzej Antosiewicz

**Affiliations:** 1 Department of Health and Natural Sciences, Gdansk University of Physical Education and Sport, 80-336 Gdansk, Poland; 2 Department of Bioenergetics and Physiology of Exercise, Medical University of Gdansk, 80-211 Gdansk, Poland; 3 Department of Athletics, Strength and Conditioning, Poznan University of Physical Education, 61-871 Poznan, Poland; 4 Department of Medical Biology and Genetics, Faculty of Biology, University of Gdansk, 80-308 Gdansk, Poland; 5 Faculty of Tourism and Recreation, Department of Health Promotion, Gdansk University of Physical Education and Sport, 80-336 Gdansk, Poland; 6 Department of Physiotherapy, Gdansk University of Physical Education and Sport, 80-336 Gdansk, Poland; 7 Department of Pharmaceutical Pathophysiology, Faculty of Pharmacy, Medical University of Gdansk, 80-210 Gdansk, Poland; 8 Department of Sport, Faculty of Physical Education, Kazimierz Wielki University in Bydgoszcz, 85-064 Bydgoszcz, Poland

**Keywords:** Ferritin, HbA1c, Soluble transferrin receptor, Body iron, Serum glucose

## Abstract

**Background and aims:**

This study evaluated whether stored iron determines the adaptive response induced by Nordic walking (NW) training combined with 10 hours’ time-restricted eating (TRE) in older adults.

**Trial design and methods:**

Twenty-four participants underwent 12-week NW training supported by 10 h of TRE. The group was divided due to baseline ferritin concentration low < 75 ng/ml (LF) and high level ≥ 75 ng/ml (HF). Body composition, physical fitness and blood collection were assessed at baseline and post-intervention.

**Results:**

NW + TRE induced a statistically significant decrease in ferritin levels in all participants (*p* = 0.01). Additionally, statistically significant intergroup differences in the LF vs. HF in the reduction of serum ferritin levels (*p* = 0.04) were observed. The procedure NW + TRE diminished HbA1c levels (*p* < 0.01) and glucose in all participants (*p* = 0.05). The range of HbA1c drop was more pronounced among those participants who experienced a greater decrease in the stored iron (*p* = 0.04, ${\eta }_{p}^{2}$=0.17, F=4.59). Greater changes in body weight and percent of body fat were recorded in the HF group (for both *p*<0.01).

**Conclusion:**

Body iron stores determine the effects of a 12-week NW + TRE intervention on serum ferritin. The changes in HbA1c are more pronounced in subjects with a higher decrease in serum ferritin.

**Trial registration:**

All experimental protocols were approved by the Bioethical Committee of the Regional Medical Society in Gdansk, Poland (NKBBN/330/2021) according to the Declaration of Helsinki. We confirm that all methods were carried out in accordance with relevant guidelines and regulations. The trial was registered as a clinical trial (NCT05229835, date of first registration: 14/01/2022, direct link: https://classic.clinicaltrials.gov/ct2/show/NCT05229835). Informed consent was obtained from all subjects.

## Introduction

### The beneficial effects of exercise on metabolism are undeniable, yet they remain far from fully understood

Adaptive changes induced by exercise might be modified by different factors like dietary supplementation, cold therapy etc. [1]. One of the important outcome of exercise is a reduction of systemic inflammation and changes in iron metabolism which are accompanied with ageing (inflammaging) [2].

Iron is an essential microelement that participates in most living organisms’ processes, such as respiration, DNA and RNA synthesis, oxygen transport, and collagen synthesis [3]. However, iron overload is a risk factor in several diseases, e.g. heart attack [4], diabetes, cancer, atherosclerosis, cognitive impairment [5], and others. Hence, iron accumulation could contribute to the induction of ‘inflammaging’. Accordingly, phlebotomy effectively reduces body iron stores, attenuates cancer risk, and improves insulin sensitivity [6, 7].

Iron is toxic because it stimulates the formation of reactive oxygen species, e.g. via the Fenton reaction. Ferritin is the main iron storage protein that protects cells from iron toxicity [8]. An increase in cell iron level leads to increased ferritin synthesis for storing excess iron and a decreased expression of transferrin receptor (TfR), the protein responsible for iron transport into the cell [8]. Conversely, if intracellular iron stores decrease because of its increased consumption or export, ferritin expression is downregulated, and that of TfR is upregulated to ensure adequate cellular iron status. Thus, iron toxicity is controlled up to a certain point, as its metabolism and storage in ferritin are tightly controlled. However, iron metabolism can be altered by exogenous factors. For instance, stress activates specific protein kinases, leading to ferritin degradation and iron-dependent oxidative stress [9]. The body’s iron stores must be controlled because it is impossible to avoid stress. Understanding the underlying mechanism and factors responsible for iron accumulation in the body is crucial for understanding the pathomechanism of several diseases.

Several non-genetic factors, such as ethanol, tea, coffee consumption, blood donation and exercise, positively or negatively influence iron accumulation [10]. For instance, regular exercise regulates iron metabolism, and a reduction of body iron stores upon exercise is reported by most studies [11, 12]. Nevertheless, some studies demonstrate no changes in serum ferritin levels upon exercise. Conversely, immobilisation of skeletal muscle or impaired insulin signalling led to iron accumulation [13].

Recently, it has been suggested that the time at which food is consumed during the day affects body composition, glucose and lipid metabolism, inflammation, sleep, and overall health [14]. Although the quality and quantity of nutrition certainly impact body iron stores, no data concerning specific effects on iron metabolism have been published [15]. According to the literature, time-restricted eating (TRE), i.e. limiting the food consumption period to 8–10 h a day, effectively improves the metabolism and reduces the risk of many diseases [16]. A report published by Parr and co-workers recently revealed that 9 h TRE improved daily measures of glycaemic control in people with T2D, visible in continuous glucose monitors [17]. Implementing TRE can give different results and not always impact the beneficial effects of exercise [18]. Among diverse forms of exercise improving glycaemic control like the recently popular interval training [19], Nordic walking (NW) training belongs to effective and safe forms of exercise. It reduces iron stores, oxidative stress, endogenous nuclear high-mobility group box 1 levels [20], and also myostatin levels [21]. The observed effects appear to be modulated by many factors, including vitamin D status, individual training experience, and body iron stores [22]. For example, exercise-induced decrease in serum myostatin levels is inversely associated with baseline ferritin levels [22]. This data clearly indicate that iron status could determine the body’s response to exercise in human.

We hypothesised that the amount of stored iron could modify the changes in metabolism generally induced by exercise combined with TRE. In the current study, we aimed to examine whether the NW plus TRE-induced changes in iron stores will be related to changes in serum glucose and HbA1c concentration in individuals aged 60 years and older. This age group is characterized by elevated iron stores much more often than iron deficiency [23].

## Materials and methods

### Study participants

**Fig. 1:**
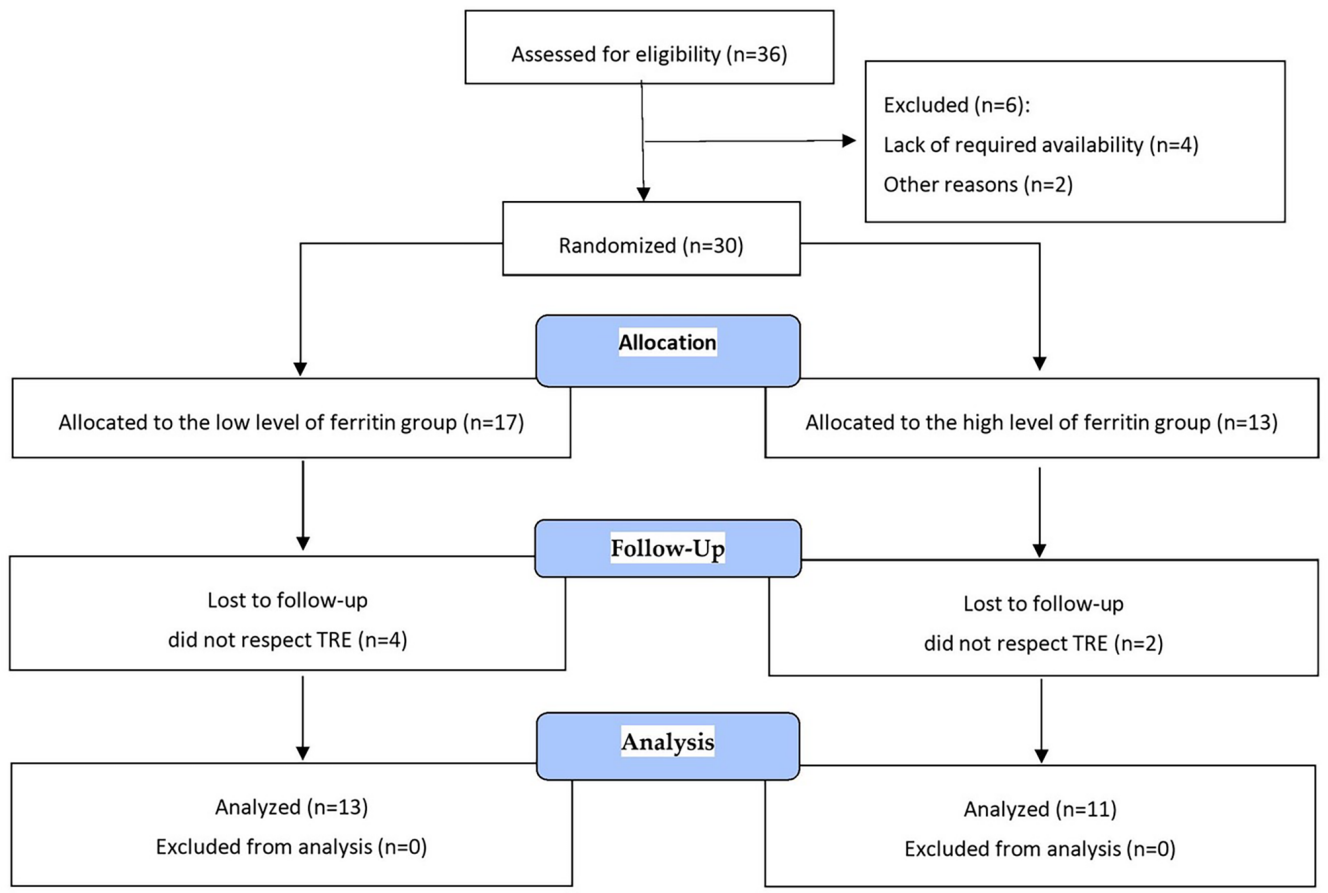
Flow diagram of the study

Thirty-six individuals responded to the publicly advertised invitation to the study. All subjects underwent a medical check-up prior to the experiment. Those with uncontrolled hypertension (diastolic blood pressure over 100 mmHg), a history of cardiac arrhythmia, cardio-respiratory disorders, and orthopaedic problems were excluded from the study. Six participants dropped out after the start of the study because of a lack of availability (*n* = 4) or for unknown reasons (*n* = 2). After allocation to parallel groups (allocation ratio 1:1), six participants were lost to follow-up during the intervention because they did not follow TRE’s 10 h eating period. In the end, twenty-four individuals (aged 70.3 ± 7.68 years) participated in the study (Fig. 1). Of these, at the beginning of the intervention, thirteen participants had low ferritin levels (LF < 75 ng/ml), and eleven had high ferritin levels (HF ≥ 75 ng/ml). The participants were divided into two walking groups to adjust the walking pace to their exercise capacity, regardless of the initial ferritin levels. The total distance performed during the 12 weeks of training was 69.39 km and 59.3 km, accordingly. The attendance rate was 86.7 ± 9%.

### Ethics statement

All experimental protocols were approved by the Bioethical Committee of the Regional Medical Society in Gdansk, Poland (NKBBN/330/2021) according to the Declaration of Helsinki. We confirm that all methods were carried out in accordance with relevant guidelines and regulations. The trial was registered as a clinical trial (NCT05229835, date of first registration: 14/01/2022, direct link: https://classic.clinicaltrials.gov/ct2/show/NCT05229835). Informed consent was obtained from all subjects.

Before commencing the training and testing, the subjects received a verbal description of the study. All the participants signed written informed consent. Additionally, ethics approval was obtained for the referral of participants to their family physician upon detection of any abnormal pathology results and review by the study medical officer. The biological sample collection were performed at Medical University of Gdansk.

### Exercise protocol

Before the start of the main experiment, all participants took part in a meeting on the training procedure (a familiarisation stage) and lectures with dietary recommendations. The training schedule was based on a published procedure [11, 24] and consisted of 3 training units/week. Each training unit took place in the morning and involved performing the main session of NW training as follows: 10 min warm-up, 45–55 min NW, and 10 min cool-down at 60–70% intensity of the maximum heart rate. Professional trainers (2 specialists) supervised each training session in two fitness-level groups, based on results of 2000 m walking test. Weekly activity data were collected using each participant’s sport-tester device for current cardiovascular control (Vantage M, Polar Electro Oy, Finland).

**Fig. 2:**
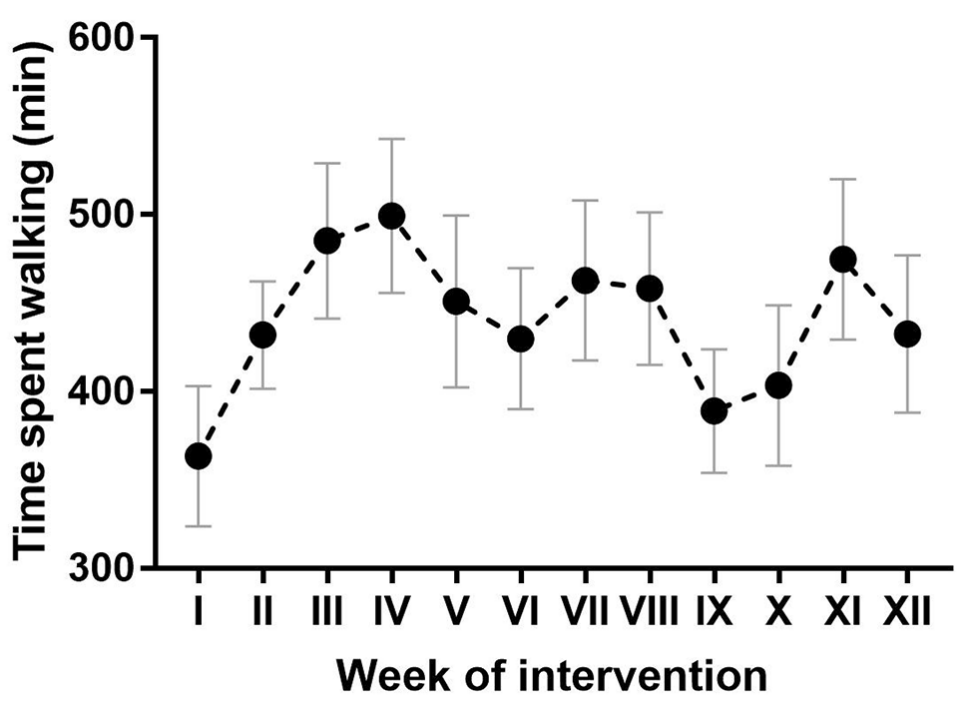
Overview of the participants’ activity during the NW + TRE intervention

The participants’ training is summarised in Table 1; Fig. 2.

**Table 1:** Assumptions of the training

Training week	I	II	III	IV	V	VI	VII	VIII	IX	X	XI	XII	
Measurment before intervention	N	NM	T1	T1	T1	T1	T1	T1	T1	T1	T1	T1	Measurment after intervention
	N	NM	TM	TM	TM	TM	TM	TM	TM	TM	TM	G	
	N	NM	T2	T2	T2	T2	T2	T2	T2	T2	T2	Z	
Intensity (%)	**30**	**30**	**40**	**40**	**40**	**50**	**50**	**50**	**60**	**60**	**70**	**70**	

Note: N - teaching the technique of walking NW, NM - teaching training methods, TM - walking test, T1 - training 1 using the repetitive method (1000 m segments), T2 - training 2 using the continuous method, G - outdoor games and activities, Z − 2000 m competition

The 12 weeks of exercise (36 training units) were divided into three microcycles (6:24:6 training units). At the beginning of the first period, proper walking technique with poles was demonstrated and then practiced under the supervision of professional trainers. The first microcycle (six training units) aimed to practice the preliminary NW components to increase the basic functional efficiency of shoulder girdle muscles, including mobility and flexibility improvement of chest muscles and shoulder joints. The second microcycle (24 training units) was the most essential component of the exercise program since all participants due to improved technique were able to cover longer training distance (expressed in km walked) and higher intensity of the exercise. Muscle-strengthening exercises were performed following every training unit. The last microcycle (six training units) was an attempt to raise the endurance level by intensifying activity and walking at the fastest possible pace. In addition, strength exercises, mainly of the back muscles, were obligatory for all participants.

### Time-restricted eating

All participants were informed about the TRE regime’s general rules, assuming a 10 h food intake window per day, followed by 14 h of fasting. The participants followed a balanced diet and adhered to healthy eating practices, i.e. food intake at regular time intervals and proper hydration. None of the participants followed a low-iron diet, i.e. vegetarian or vegan. The participants were advised not to change their original dietary habits and to complete their daily time of eating table according to the TRE plan.

### Measurement of body composition and physical fitness

Each participant’s body mass and composition were determined using a multi-frequency impedance plethysmograph body composition analyser. The body mass was determined after overnight fasting (minimum 12 h). The impedance of body parts (trunk, arm, and leg segments) was measured at six different frequencies (1, 5, 50, 250, 500, and 1000 kHz) using an eight-polar tactile-electrode (Inbody720, Biospace, USA).

A senior fitness test for examining elderly people developed by Rikli [25] was used to determine the functional fitness of the participants. The test consists of six items: [1] 30 s chair stand– the assessment quantifies the quantity of sit-to-stand repetitions accomplished within a 30-second timeframe, serving as a metric for evaluating lower extremity muscular strength; [2] arm curl– the evaluation discerns upper body strength by quantifying the number of bicep curls performed within a 30-second interval; [3] chair sit-and-reach– measurement of the distance between fingertips and toes while bending forward in a sitting position on a chair, it reflects lower body flexibility [4] back scratch - the examination measures the linear gap between the middle finger during the execution of an overhead reach with one hand over the shoulder and the other hand reaching up the back. The outcome of this test serves as an indicator of upper body flexibility; [5] 8-foot up-and-go– the assessment evaluates agility and dynamic balance by quantifying the duration, in seconds, for older adults to execute a sequence comprising standing up, walking 8 feet, performing a turn, and returning to a seated position; [6] 2 min step - examination appraises aerobic endurance by measuring the number of steps completed within 2 min. The items were tested in this order, with 1 min rest between them. Before each item was tested, the evaluator demonstrated the exercise, and the participant had an attempt at familiarisation, except for the 2 min step test, which the subjects performed only once. The test was conducted twice after the recruitment and after 12 weeks of intervention.

### Assessment of the handgrip

Saehan hydraulic dynamometer (Saehan Corporation, 973, South Korea) was used to measure grip strength. First, the right hand was tested and then the left hand, with three repetitions for each hand. Accessories, such as watches, bracelets, and rings, were removed from both arms before the start of testing. Participants were instructed to maximally contract the tested hand for 3 s in each test. There was a rest period of 30 s between each test and a rest period of 2 min between the testing of each hand. During the test, the participants were comfortably seated in an armless chair, with feet flat on the floor and hip and knee positioned at approximately 90 degrees of flexion. The shoulder of the tested limb was abducted and neutrally rotated, elbow flexed at 90 degrees, with the forearm and wrist in a neutral position between 0 and 30 degrees of extension and from 0 to 15 degrees of adduction. The hand that was not tested rested on the leg on the same side. The same examiner evaluated all participants.

### Maximal oxygen uptake ($\dot{V}$O_2_max) determination in 2000 m walking test

Endurance was measured using the 2000 m walking test (UKK Walk test). The test consisted of brisk walking at an even pace. The test was carried out on a racetrack in a sports hall at 18 °C (Academy of Physical Education and Sport, Gdansk, Poland) to exclude variables related to the weather conditions and surface. The participants covered 10 loops, each 200 m long, with gentle curves so that they would not have to slow down to take turns. Before the test, the participants performed a warm-up consisting of dynamic exercises stimulating them to walk briskly. They performed the test wearing sports clothes and shoes. During the test, the subjects were mobilised to march as fast as possible; however, running was not allowed. The maximum oxygen uptake was calculated using a 2000 m waking test [26].

### Blood analysis

Biochemical blood analysis was performed at baseline (i.e. 1 day before the start of the intervention) and then on the first day immediately after the 12-week NW + TRE program. A blood sample (4 ml) was drawn into a blood tube (Becton Dickinson, USA) from the arm vein of each participant by a trained phlebotomist, between 7 and 8 AM, following an overnight fasting. Blood samples were directly analysed using BIOSYSTEMS apparatus (BIOSYSTEMS S.A., Spain) to determine the red blood cell count (RBC, 10^6^⋅µl^− 1^), haematocrit (Ht, %), and blood haemoglobin (Hb) levels (g⋅dl^− 1^). The average intra-assay coefficient of variability was < 10% for all measurements. Glucose, glycated haemoglobin (HbA1c), lipid profile (total cholesterol– TC, low-density lipoproteins cholesterol– LDL-C, high-density lipoproteins cholesterol– HDL-C, and triglycerides– TG) and aminotransferase levels (ALT and AspAT) were determined using a Cobas 6000 analyser (ROCHE, [France]), and serum ferritin levels were determined by using SYSMEX XE 2100 ([Sysmex co, Japan]).

Body iron was calculated from the ratio of soluble transferrin receptor to serum ferritin (R/F) as follows: body iron (mg/kg)= -[log(R/F) − 2.8229]/0.1207 [27].

The systemic immune-inflammation index (SII) was calculated using the following formula: SII = P*N/L. where P, N, and L refer to the peripheral platelet, neutrophils, and lymphocyte counts, respectively [28].

### Statistical analysis

Statistical analysis was performed using Statistica 13.1 software. Graphs were created in GraphPrism 7 software. All values are expressed as the mean ± standard deviation (SD). The Shapiro-Wilk test was used to assess the homogeneity of dispersion from a normal distribution. The Brown-Forsythe test was used to evaluate the homogeneity of variance. First, the effectiveness of the NW + TRE intervention was analysed. A paired t-test analysis was performed to identify significant differences for homogenous results. For heterogeneous results, the Wilcoxon signed-rank test was used. Next, the influence of ferritin baseline levels was assessed by comparing changes in LF and HF groups. For homogenous results, analysis of variance (ANOVA) for repeated measures and the post-hoc Tukey’s test for unequal sample sizes were performed to identify significant differences. For heterogeneous results, ANOVA Friedman’s test and the Dunn-Bonferroni post-hoc test were used. The effect size (partial eta squared, ${\eta }_{p}^{2}$) was also calculated, with ${\eta }_{p}^{2}$≥0.01 indicating a small effect; ≥0.059 indicating a medium effect, and ≥ 0.138 indicating a large effect [29]. The significance level was set at *p* < 0.05. The relationships between variables were evaluated using Spearman’s correlation coefficient. The sample size was predetermined by using a power calculation in G∗power v3.1.9.7 software. In the current study, iron levels were the main factor for determining the calculated sample size based on the partial eta-squared effect size of 0.06. Based on a priori power analysis for family F tests in ANOVA repeated measures, within-between (group x time) interaction, at least 12 participants were included in each group (α = 0.05, 1–β = 0.8, f = 0.25, r_rm_=0.85; ε = 1).

## Results

After 12 weeks of NW training combined with daily 10 h TRE (NW + TRE), a statistically significant decrease in body weight (mean change: − 1.82 kg, *p* < 0.01) and body fat mass and percentage (–2.5 kg and − 1.8%, respectively, *p* < 0.01) was noted. Further, the training program effectively increased the functional fitness of the participants. Significant changes in all measurements except for the chair sit-and-reach on the right side of the body were observed (Table 2). Unexpectedly, the strength of the handgrip decreased (mean change for the left hand: − 1.47 kg, *p* = 0.03; and for the right hand: − 2.44 kg, *p* < 0.01).

**Table 2:** Changes in the anthropometric parameters and functional fitness after 12 weeks of NW + TRE intervention

	All participants (*n* = 24)			*p*	LF (*n* = 13)	HF (*n* = 11)	*p*
	PRE	POST	Δ (95%CI)		Δ (95%CI)	Δ (95%CI)	
**Anthropometric parameters**							
**BW (kg)**	71 ± 13.1	69.83 ± 12.78	–1.82 (–2.39; − 1.25)	**< 0.01**	–1.72 (–2.28; − 1.16)	–1.94 (–3.08; − 0.79)	0.70
**BMI (kg·m** ^**–2**^ **)**	26.3 ± 3.87	25.62 ± 4.05	–0.78 (–1.33; − 0.24)	**< 0.01**	–0.97 (–2.02; 0.07)	–0.57 (–1.02; − 0.13)	0.46
**Fat (kg)**	24.45 ± 8.39	22.25 ± 8.3	–2.5 (–3.71; − 1.28)	**< 0.01**	–1.3 (–1.61; − 0.99)	–3.8 (–6.26; − 1.34)	**0.03**
**Fat %**	33.1 ± 8.35	31.39 ± 8.74	–1.8 (–2.5; –1.1)	**< 0.01**	–1.13 (–1.63; − 0.62)	–2.53 (–3.86; − 1.19)	**0.03**
**FFM (kg)**	47.05 ± 8.57	47.58 ± 9.27	0.16 (–0.41; 0.72)	0.57	–0.42 (–0.92; 0.09)	0.78 (–0.22; 1.78)	**0.02**
**TBW (kg)**	34.62 ± 6.34	34.99 ± 6.84	0.1 (–0.31; 0.5)	0.63	–0.32 (–0.68; 0.05)	0.55 (–0.18; 1.27)	**0.02**
**SMM (kg)**	25.68 ± 5.29	26.02 ± 5.59	0.18 (–0.16; 0.52)	0.28	–0.17 (–0.48; 0.13)	0.54 (–0.05; 1.12)	**0.03**
**VFA (cm** ^**2**^ **)**	137.11 ± 31.52	134.24 ± 32.21	–2.93 (–5.45; − 0.41)	**0.02**	–2.07 (–3.79; − 0.34)	–3.87 (–9.28; 1.53)	0.47
**Functional fitness**							
**Arm curl (n) left**	23.33 ± 6.21	28.3 ± 5.81	5.95 (3.87; 8.03)	**< 0.01**	5 (2.33; 7.67)	7.11 (3.32; 10.91)	0.30
**Arm curl (n) right**	23.08 ± 6.11	28.6 ± 6.37	6.8 (4.67; 8.93)	**< 0.01**	6 (3.17; 8.83)	7.78 (3.92; 11.64)	0.40
**Back scratch (cm) left**	–9.71 ± 9.44	–7.25 ± 8.39	4.15 (2.38; 5.92)	**< 0.01**	3.36 (1.23; 5.49)	5.11 (1.71; 8.52)	0.32
**Back scratch (cm) right**	–8.17 ± 9.16	–4.2 ± 7.43	4.6 (3.08; 6.12)	**< 0.01**	3.73 (1.42; 6.04)	5.67 (3.49; 7.84)	0.19
**Chair sit–and–reach (cm) left**	4.38 ± 8.3	5.95 ± 6.28	2.9 (0.61; 5.19)	**0.02**	2.73 (–0.76; 6.22)	3.11 (–0.56; 6.79)	0.87
**Chair sit–and–reach (cm) right**	4.54 ± 8.74	4.95 ± 6.45	1.95 (–0.83; 4.73)	0.70	1.09 (–2.71; 4.9)	3 (–1.94; 7.94)	0.49
**8 Foot up–and–go (s)**	5.47 ± 0.63	4.92 ± 0.75	–0.58 (–0.95; − 0.21)	**< 0.01**	–0.59 (–1.1; − 0.09)	–0.57 (–1.23; 0.1)	0.94
**Chair stand (n)**	17.95 ± 3.01	22.45 ± 3.07	4.78 (2.81; 6.75)	**< 0.01**	5.22 (2.71; 7.74)	4.33 (0.71; 7.96)	0.65
$\dot{V}$ **O**_**2max**_ **(ml/kg/min)**	31.68 ± 4.31	34.98 ± 4.18	3.31 (2.51; 4.1)	**< 0.01**	2.63 (1.51; 3.75)	4.13 (3.07; 5.2)	**0.04**
**HG right (kg)**	32.32 ± 7.98	29.26 ± 5.99	–2.44 (–3.99; − 0.89)	**< 0.01**	–2.34 (–3.37; − 1.3)	–2.56 (–6; 0.89)	0.89
**HG left (kg)**	29.77 ± 8.54	27.42 ± 6.53	–1.47 (–2.8; − 0.14)	**0.03**	–1.16 (–2.79; 0.47)	–1.81 (–4.31; 0.68)	0.62

Values are means ± SD. PRE– measurement before intervention; POST– measurement before intervention; The statistical significance level was calculated using nonparametric analysis (italics) or parametric analysis (regular font). BW, body weight; BMI, body mass index; Fat, fat mass; FFM, free fat mass; TBW, total body water; SMM, skeletal muscle mass; VFA, visceral fat area; HG, hand grip; LF, low-ferritin group, < 75 ng/ml at the beginning of the intervention; HF, high-ferritin group, ≥ 75 ng/ml at the beginning of the intervention; Δ, the mean difference between the measured value after and before the intervention; 95% CI, 95% confidence interval of differences between the study groups at baseline

Considering the baseline ferritin levels, the decrease in fat percentage [LF: − 1.13%, CI=.

–1.63; − 0.62); HF: − 2.53%, CI=(–3.86; − 1.19); *p* = 0.03] and increase in the $\dot{V}$O_2max_ [LF: 2.63 ml/kg/min, CI=(1.51; 3.75); HF: 4.13 ml/kg/min, CI=(3.07; 5.2); *p* = 0.04] were significantly more pronounced in the HF group than those in the LF group (Table 3).

**Table 3:** Metabolic changes concerning baseline iron status after 12 weeks of NW + TRE intervention

	All participants (*n* = 24)			*p*	LF (*n* = 13)	HF (*n* = 11)	*p*
	PRE	POST	Δ (95%CI)		Δ (95%CI)	Δ (95%CI)	
**Indicators of iron metabolism**							
***Iron (µg/dl)***	98.83 ± 25.48	95.21 ± 26.75	–3.63 (–17.2; 9.95)	0.75	3.15 (–12.85; 19.16)	–11.64 (–36.82; 13.55)	0.27
**Ferritin (ng/ml)**	113.3 ± 90.67	96.42 ± 78.64*	–16.88 (–28.85; − 4.91)	**0.01**	–6.05 (–13.98; 1.89)	–29.68 (–53.98; − 5.38)	**0.04**
**Tf (g/l)**	2.76 ± 0.28	2.73 ± 0.28	–0.02 (–0.1; 0.05)	0.52	–0.03 (–0.14; 0.07)	–0.01 (–0.13; 0.11)	0.75
**Body iron (mg/kg)**	38.99 ± 2.69	38.07 ± 2.77	–0.93 (–1.41; − 0.44)	**< 0.01**	–0.73 (–1.49; 0.02)	–1.15 (–1.86; − 0.45)	0.39
**sTfR (mg/l)**	1.2 ± 0.29	1.32 ± 0.37	0.12 (0.03; 0.22)	**0.01**	0.1 (–0.02; 0.22)	0.15 (–0.02; 0.33)	0.59
**Haematological indicators**							
**Hb (g/dl)**	13.98 ± 1.06	13.62 ± 0.97*	–0.36 (–0.52; − 0.2)	**< 0.01**	–0.33 (–0.59; − 0.07)	–0.4 (–0.61; − 0.19)	0.66
***HbA1c (mmol/mol)***	42.08 ± 5.46	39.17 ± 5.01*	–2.92 (–3.79; -2.05)	**< 0.01**	–2.31 (–3.31; − 1.31)	–3.64 (–5.21; − 2.07)	0.12
***HbA1c (%)***	5.98 ± 0.5	5.73 ± 0.47*	–0.25 (–0.26; − 0.14)	**< 0.01**	–0.22 (–0.3; − 0.13)	–0.3 (–0.45; − 0.15)	0.27
**RBC (T/l)**	4.58 ± 0.35	4.49 ± 0.35*	–0.09 (–0.19; − 0.1)	**< 0.01**	–0.09 (–0.18; − 0.01)	–0.09 (–0.17; 0)	0.88
***Ht (%)***	42.05 ± 2.6	41.1 ± 2.66	–0.96 (–1.95; − 1.08)	**0.01**	–1.12 (–2.05; − 0.2)	–0.76 (–1.61; 0.08)	0.54
**MCV (fl.)**	92.03 ± 3.19	91.72 ± 3.34	–0.32 (–2.45; − 1.36)	0.38	–0.61 (–1.75; 0.54)	0.03 (–1.03; 1.09)	0.39
**MCH (pg)**	30.56 ± 1.27	30.38 ± 1.31	–0.18 (–0.74; − 0.41)	0.12	–0.09 (–0.45; 0.27)	–0.27 (–0.57; 0.03)	0.42
**MCHC (g/dl)**	33.23 ± 0.97	33.13 ± 0.64	–0.1 (–1.07; − 0.59)	0.54	0.1 (–0.42; 0.62)	–0.33 (–0.72; 0.06)	0.18
**WBC (G/l)**	5.53 ± 1.23	5.77 ± 1.2	0.24 (0.45;0.82)	0.06	0.37 (0.11;0.64)	0.09 (–0.39;0.57)	0.25
**Biochemical indicators**							
***SII (cells/l)***	414.42 ± 211.46	370.43 ± 191.39	–43.99 (–107.6; 19.61)	0.41	–55.68 (–148.86; 37.5)	–30.18 (–132.65; 72.28)	0.69
***AspAT (U/l)***	23.58 ± 5.56	24.5 ± 5.44	0.92 (–0.8; 2.63)	0.28	2.62 (0.17; 5.06)	–1.09 (–3.22; 1.04)	**0.02**
**ALT (U/l)**	24.17 ± 10.66	24.29 ± 10.07	0.12 (–2.06; 2.31)	0.91	2.77 (–0.11; 5.65)	–3 (–5.57; − 0.43)	**< 0.01**
**Glucose (mg/dl)**	101.83 ± 24.06	98.42 ± 21.84	–3.42 (–6.82; − 0.01)	**0.05**	–3.77 (–8.2; 0.66)	–3 (–9.19; 3.19)	0.82
**TC (mg/dl)**	197.17 ± 45.32	196.17 ± 44.77	–1 (–10.87; 8.87)	0.84	0.38 (–15.26; 16.03)	–2.64 (–16.84; 11.56)	0.76
**TG (mg/dl)**	111.42 ± 44.4	107.58 ± 58.91	–3.83 (–20.81; 13.15)	0.64	–1.77 (–27.75; 24.21)	–6.27 (–32.2; 19.65)	0.79
**HDL-C (mg/dl)**	62.42 ± 15.44	61.42 ± 13.15	–1 (–4.52; 2.52)	0.56	–2.77 (–7.9; 2.36)	1.09 (–4.3; 6.48)	0.27
**LDL-C (mg/dl)**	112.5 ± 40.99	113.21 ± 39.2	0.71 (–7.58; 9)	0.86	3.46 (–9.13; 16.06)	–2.55 (–15.01; 9.92)	0.47

Values are means ± SD. PRE– measurement before intervention; POST– measurement before intervention; The statistical significance level was calculated using nonparametric analysis (italics) or parametric analysis (standard font). * significantly different from baseline value. Tf, transferrin, sTfR, serum soluble transferrin receptor; Hb, haemoglobin; HbA1c, glycated haemoglobin; RBC, red blood cell; Ht, haematocrit; MCV, mean corpuscular volume; MCH, mean cell haemoglobin; MCHC, mean cellular haemoglobin concentration; WBC, white blood cells; SII, systemic immune–inflammation index; AspAT, aspartate aminotransferase; ALT, alanine aminotransferase; TC, total cholesterol; TG, triglycerides; HDL-C, high-density lipoprotein; LDL-C, low-density lipoprotein; LF, low-ferritin group, < 75 ng/ml at the beginning of the intervention; HF, high-ferritin group, ≥ 75 ng/ml at the beginning of the intervention; Δ, the mean difference between measured value after and before the intervention; 95% CI, 95% confidence interval of differences between the study groups at baseline

After the NW + TRE intervention, a statistically significant decrease in ferritin [Δ= − 16.88 ng/ml, CI=(–28.85; − 4.91), *p* = 0.01] and body iron levels (Δ=–0.93 mg/kg, CI=(–1.41; − 0.44), *p* < 0.01] was noticed, while serum soluble transferrin receptor (sTfR) [Δ = 0.12 mg/l; CI=(0.03;0.22), *p* = 0.01] (Table 3) increased in both groups. Differences in the ferritin response between the groups depended on the ferritin levels at baseline [LF: Δ= − 6.05 ng/ml, CI=(–13.98; 1.89); HF: Δ=–29.68 ng/ml, CI=(–53.98; − 5.38), *p* = 0.04].

**Fig. 3:**
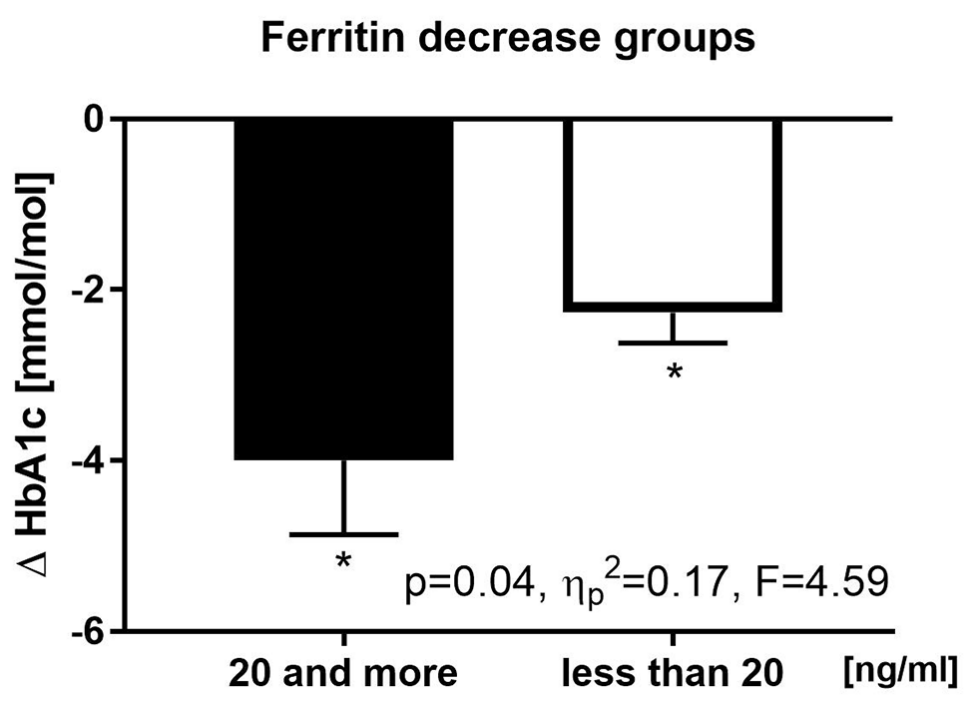
Effects of NW + TRE on HbA1c in subgroups with different changes in serum (by repeated-measures analyses of variance with post-hoc tests,${\eta }_{p}^{2}$– the effect size, F– value in repeated measures ANOVA * *p* < 0.05). The group where ferritin decreased by more than 20ng/ml (*n* = 9) and less than 20ng/ml (*n* = 15)

In addition, changes in the HbA1c (glycated haemoglobin; Δ=-2.92 mmol/mol, CI=(–3.79; − 2.05), *p* < 0.01] and glucose levels [Δ=–3.42 mmol/mol, CI=(–6.82; − 0.01), *p* = 0.05] were observed. The change in HbA1c levels was greater in the HF group than that in the LF group (HF: − 3.64 mmol/mol vs. LF: − 2.31 mmol/mol) but did not reach statistical significance (*p* = 0.12). Of note, in participants whose ferritin levels dropped after the intervention by more than 20 ng/ml, the decrease in HbA1c levels was significantly higher than that in whom the decrease was smaller (Fig. 3).

Considering the systemic immune–inflammation index (SII), the SII decrease was significantly greater among individuals with a baseline index above 410 [Δ= − 120.8 cells/l, CI=(–260.61; 19.01] than in those with below [Δ = 10.87 cells/l, CI=(–33.54; 55.28), *p* = 0.03], and was accompanied by a decrease in glucose levels [Δ=–7.7 mg/dl, CI=(–12.75; − 2.65)] compared to that with SII lower than 410 [–0.36 mg/dl, CI=(–4.7; 3.98); *p* = 0.02].

## Discussion

A previously published study has shown that NW endurance training reduces body iron levels, and this effect was associated with reduced oxidative stress and improved physical performance [24]. Contrary, it is also known that other factors can limit beneficial changes induced by training. Thus, the main goal of the present study was to evaluate whether changes in iron metabolism induced by exercise combined with TRE depend on the baseline iron stores (serum ferritin) and whether these changes are associated with shifts in blood glucose and Hb1Ac levels.

Obtained data indicated that 12 weeks of NW training combined with TRE altered the body composition, including a significant decrease in body mass, body fat mass content, and body mass index (BMI). Of note, these effects were not apparent in our previous study, in which NW was tested alone [24]. According to a few studies, endurance exercise and recreational activity reduce body iron stores [11, 12]. Here, we hypothesised that the effect of exercise combined with TRE on body iron stores depends on baseline iron stores. Indeed, the changes induced by the tested NW + TRE intervention depended on the baseline ferritin levels. Specifically, induced changes were more pronounced in the high-ferritin group (HF, baseline serum ferritin ≥ 75 ng/ml) than in the low-ferritin group (LF, baseline serum ferritin < 75 ng/ml). Therefore, we concluded that NW combined with TRE reduces body iron stores only in subjects with relatively high initial body iron stores.

Ferritin is a good marker of body iron stores without acute inflammation [30]. The current study calculated the body’s iron stores based on ferritin and sTfR levels. The decrease in these indicators is a beneficial effect of NW + TRE. According to many studies, high body iron stores are associated with an increased risk of several diseases. No normal value range for body iron stores has yet been established; however, the upper value for ferritin is 200–300 ng/ml [31]. Notably, the ferritin levels were within the normal range in most participants of the current study. Since several studies have pointed out that ferritin levels exceeding 60–100 ng/ml are associated with lower physical performance, higher risk of cancer and heart disease, and insulin resistance [6, 32], the observed effect of NW + TRE should be considered as very desirable.

The current study has also demonstrated the profound effect of the NW + TRE intervention on glycaemic control, i.e. fasting plasma glucose and HbA1c levels. HbA1c is a good marker of average blood sugar levels in the preceding 3 months. Hence, the HbA1c levels indicate the effect of an intervention on long-term regulation of serum glucose levels. The HbA1c levels in most participants of the current study were equal to 5.7% or higher, which indicates a pre-diabetic state [33]. Of note, a decrease of 1% in HbA1c levels has been related to a 15–20% reduction in cardiovascular disease events and a 37% reduction in microvascular complications [34–36]. In the current study, we observed a decrease of HbA1c 0.25% levels after the NW + TRE intervention. This may be expected to reduce the risk of cardiovascular disease by 5% and microvascular complications by 9%.

The underlying mechanism of the decrease in HbA1c levels upon NW + TRE is not entirely clear, but it potentially involves a reduction of body iron stores. First, reducing body iron stores can decrease oxidative stress, one of the drivers of HbA1c formation [37]. Further, it has been demonstrated that phlebotomy, a procedure that effectively reduces body iron stores, induces a decrease in HbA1c levels [38]. In the current study, the decrease in HbA1c levels after the NW + TRE intervention was similar, regardless of the baseline ferritin levels. However, in participants whose ferritin levels dropped after the intervention by more than 20 ng/ml, the decrease in HbA1c levels was significantly higher than that in whom the decrease was less profound, indicating the role of iron in this process.

Here, we demonstrated an increased concentration of serum sTfR. It is a fragment of membrane-associated TfR, a good marker for detecting iron deficiency and calculating body iron stores. Its levels reflect the rate of erythropoiesis, and an increase in sTfR usually reflects a decrease in tissue iron stores [39]. In the current study, the decreased serum ferritin levels after NW + TRE indicated decreased tissue iron levels. Hence, the observed increase in sTfR levels could be considered a physiological response to increasing demand for iron, while a decrease could mean the opposite. However, in the current study, the changes in sTfR levels were similar regardless of the baseline ferritin levels. These findings indicate that other mechanisms, in addition to iron, regulate sTfR levels. Importantly, serum sTfR levels remain stable after acute exercise or after a brief training period [40, 41].

Conversely, some studies have demonstrated a significant association between serum sTfR levels and chronic diseases. For example, elevated sTfR levels are associated with a risk of diabetes mellitus and heart failure [42, 43], and sTfR has been suggested to be a good marker of all-cause mortality [44]. Hence, it is not entirely clear whether the sTfR level increase observed in the current study in elderly individuals (the study participants) could be considered a positive change. In one study, sTfR levels were higher in athletes than in control nontrained men [40]. Hence, the changes in our trained older adults can be considered encouraging as they are similar to that observed among young athletes [40].

Together with iron deficiency, hypoxia can upregulate TfR levels. The promoter region of the TfR gene contains a hypoxia response element, which binds hypoxia-inducible factor 1 (HIF-1) [45]. Exercise-induced hypoxemia has been observed even in young endurance athletes [46]. Hence, we cannot exclude the possibility that regular exercise induces transient hypoxia in seniors, which could increase TfR expression [47]. In support of this hypothesis, the changes in sTfR levels in study participants whose ferritin levels decreased more than 20 ng/ml due to NW + TRE were the same as those in the participants with more minor changes in ferritin levels. This suggests that iron does not mediate the observed changes in sTfR levels.

**Fig. 4:**
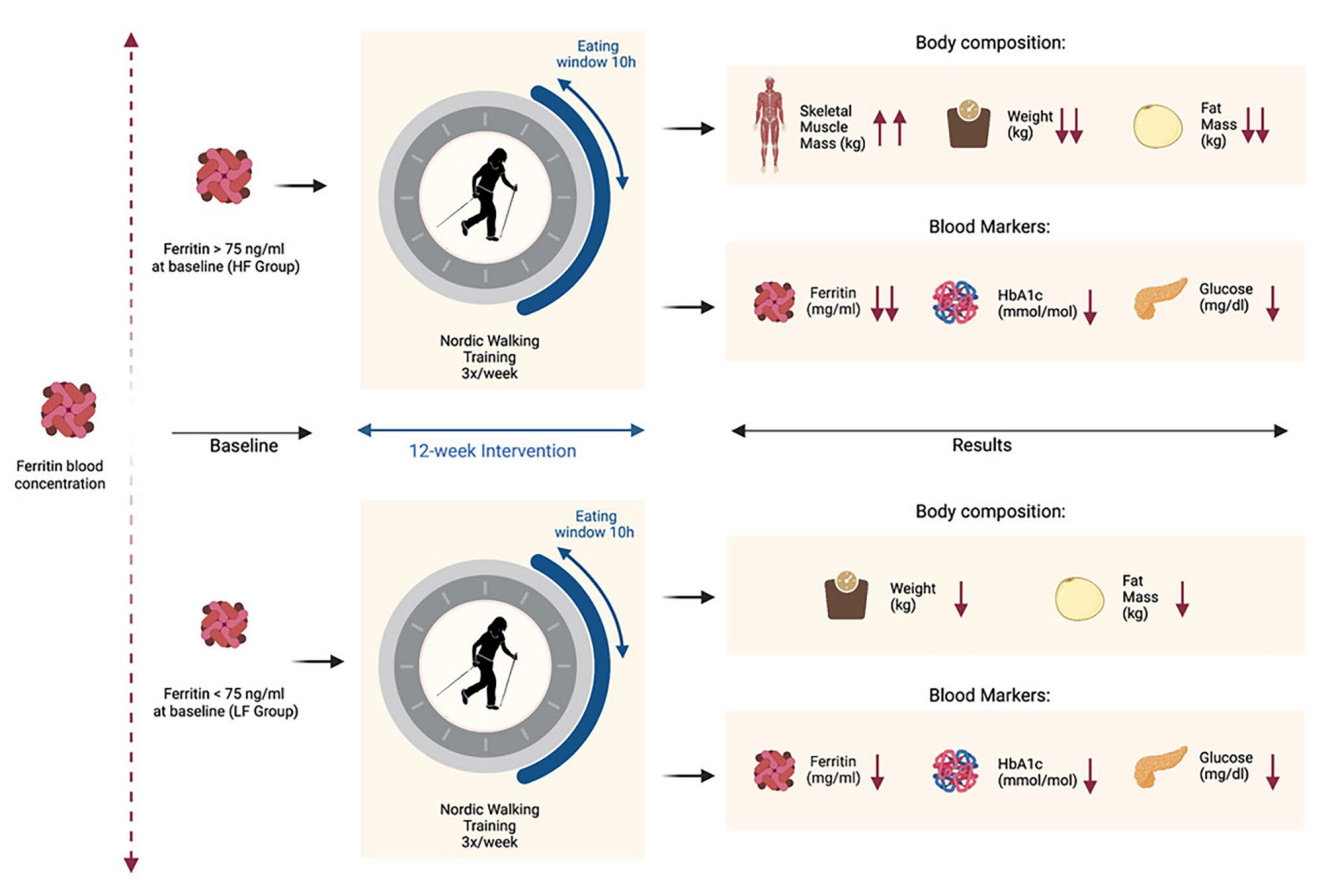
Beneficial effects of time-restricted eating (TRE) combined with Nordic Walking training (NW) are related to baseline ferritin concentration. During 12 weeks of NW + TRE, changes in ferritin, body weight, skeletal muscle mass, and fat mass were significantly higher in participants with baseline ferritin above 75 ng/ml (HF) compared to those with ferritin lower than 75 ng/ml (LF). The changes in blood glucose and HbA1c were similar in both groups (↓) -indicates that changes were significant after the intervention in all subjects, (↓↓ or ↑↑)- Indicates that the changes significantly differed from those observed in participants with serum ferritin lower than 75 ng/ml. Created with BioRender.com

## Conclusions

We here demonstrated that body iron stores determine the pro-healthy effects of NW + TRE. The intervention effectively reduced body iron stores, mainly in individuals with baseline ferritin levels above 75 ng/ml. Further, the interrelationship between changes in HbA1c and ferritin levels indicates that in addition to glucose, iron significantly contributes to haemoglobin glycation (Fig. 4).

## Data Availability

The datasets used and/or analysed for the current study are available from the corresponding author upon reasonable request.

## Abbreviations

Tf: Transferrin,sTfR,serum soluble transferrin receptor
Hb: Haemoglobin
HbA1c: Glycated haemoglobin
RBC: Red blood cell
Ht: Haematocrit
MCV: Mean corpuscular volume
MCH: Mean cell haemoglobin
MCHC: Mean cellular haemoglobin concentration
WBC: White blood cells
SII: Systemic immune–inflammation index
AspAT: Aspartate aminotransferase
ALT: Alanine aminotransferase
TC: Total cholesterol
TG: Triglycerides
HDL-C: High-density lipoprotein
LDL-C: Low-density lipoprotein
LF: Low-ferritin group,< 75 ng/ml at the beginning of the intervention
HF: High-ferritin group,≥ 75 ng/ml at the beginning of the intervention
Δ: The mean difference between measured value after and before the intervention
BW: Body weight
BMI: Body mass index
Fat: Fat mass
FFM: Free fat mass
TBW: Total body water
SMM: Skeletal muscle mass
VFA: Visceral fat area
HG: Hand grip
